# Supplementary material for: Nb/Ta systematics in arc magma differentiation and the role of arclogites in continent formation
Source: Nat Commun. 2019 Jan 16;10:235. doi: 10.1038/s41467-018-08198-3 (PMC6335430; doi:10.1038/s41467-018-08198-3)
Supplement: Supplementary file 1 — Supplementary Information [file 41467_2018_8198_MOESM1_ESM.pdf]

Nb/Ta systematics in arc magma differentiation and the role of arclogites in continent formation

Tang et al.

Supplementary Information

Supplementary Figure 1. Sample locations of the compiled arc, ocean island and small volume intracontinental igneous rocks. The map was produced using GeoMapApp (http://www.geomapapp.org).

Supplementary Figure 2. Distributions of Nb and Ta concentrations and Nb/Ta ratios in mid-ocean ridge basalts (MORB) (n = 2,264) and arc basalts (n = 1,964). The MORB data are from Gale et al[12]. The arc basalt data are compiled from GeoRoc.

Supplementary Figure 3. Comparison of Nb/Ta for six reference materials (BCR-2, BIR-1, BHVO-2, RGM-2, GSP-2 and AGV-2) measured by solution ICP-MS from this study and GeoRem preferred values or average published values[59]. The uncertainties (3.8%, 2 RSD) for our measured Nb/Ta are based on six replicates of BHVO-2 (Supplementary Data 1). A 5% uncertainty is assumed for GeoRem data.

Supplementary Figure 4. Comparison of whole rock Nb and Ta concentrations measured by solution ICP-MS and LA-ICP-MS methods. LA-ICP-MS measurements were done on lithium metaborate-fused glass discs at Rice University. The data were published at http://dx.doi.org/10.1594/IEDA/111138.

Supplementary Figure 5. Mean magma composition parameter (FM) as a function SiO_2 content in arc magmas. Arc magma composition data are compiled from GeoRoc. The error bars are 2 se and are about the same size as the markers.

Supplementary Figure 6. Rutile saturation pressure as a function of temperature and TiO_2 content in the melt. We assumed a constant magma composition parameter (FM) of 4, which is the upper bound value for arc magmas with 60−65 wt.% SiO_2 (Supplementary Figure 5).

Supplementary Figure 7. pMELTS simulation of garnet pyroxenite and peridotite decompression melting in the mantle.

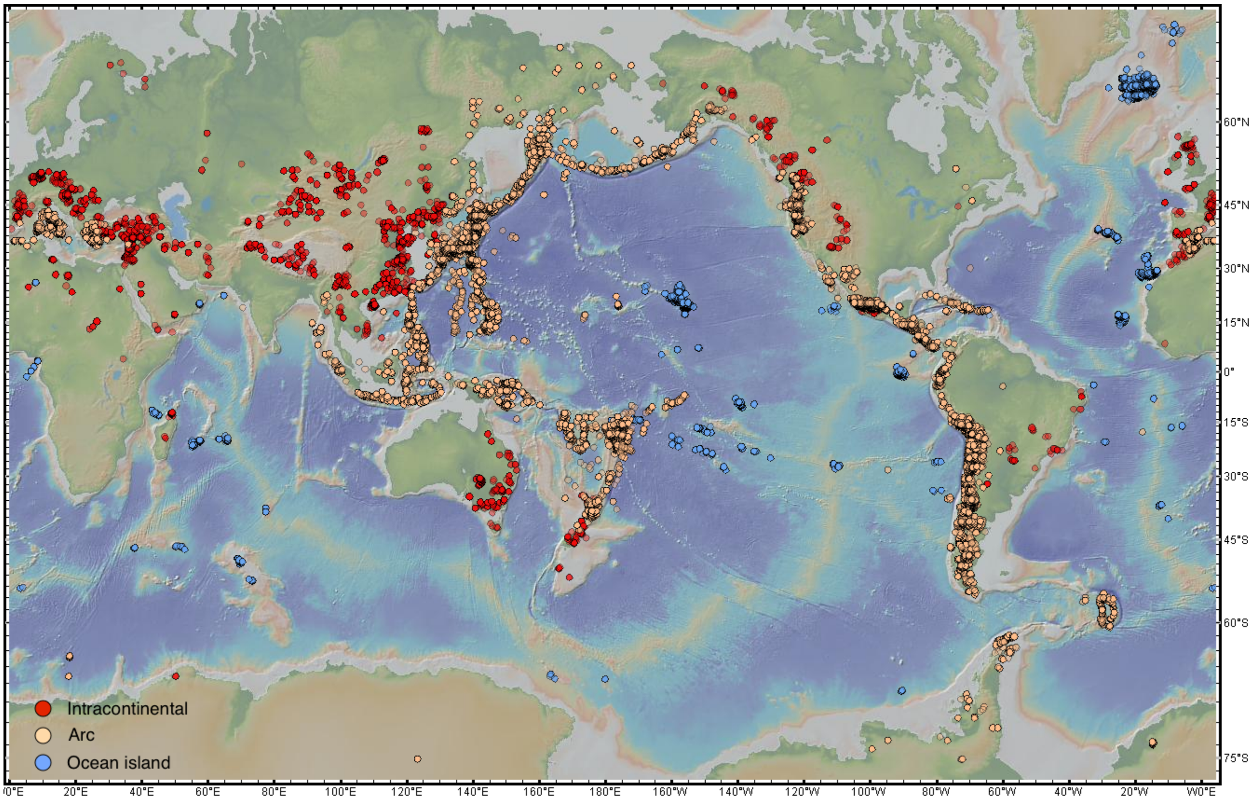

Supplementary Figure 1. Sample locations of the compiled arc, ocean island and small volume intracontinental igneous rocks. The map was produced using GeoMapApp (http://www.geomapapp.org).

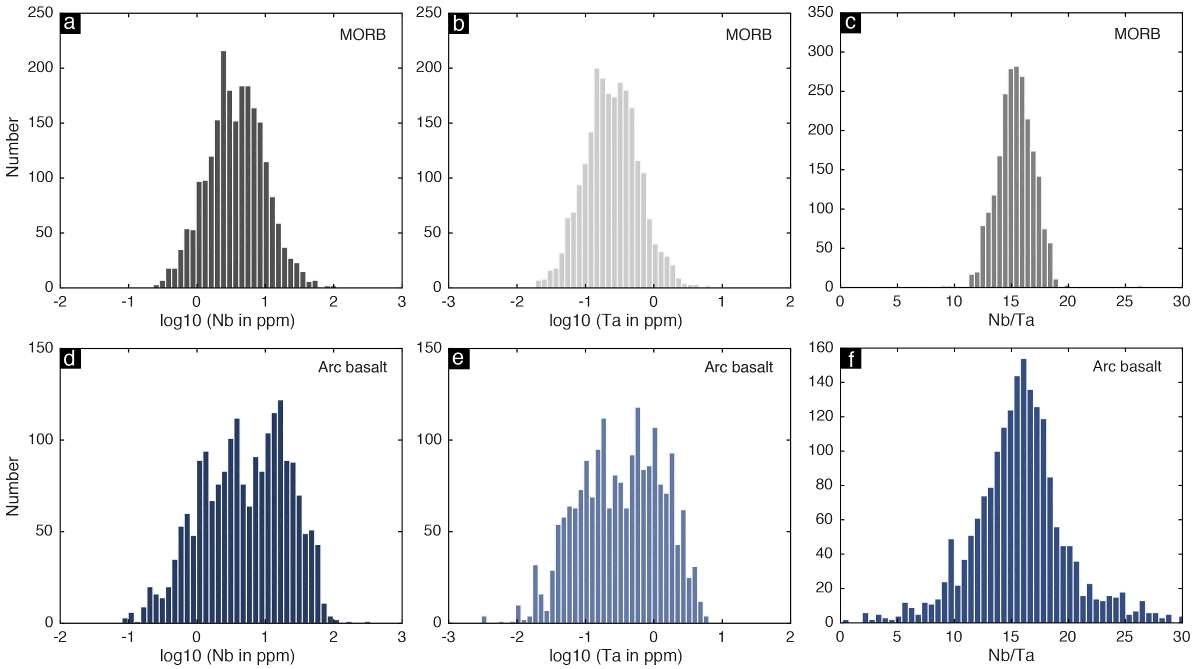

Supplementary Figure 2. Distributions of Nb and Ta concentrations and Nb/Ta ratios in mid-ocean ridge basalts (MORB) (n = 2,264) and arc basalts (n = 1,964). The MORB data are from Gale et al[12]. The arc basalt data are compiled from GeoRoc.

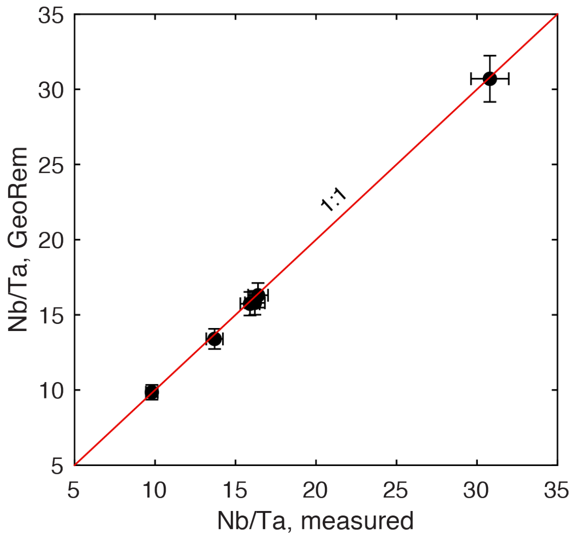

Supplementary Figure 3. Comparison of Nb/Ta for six reference materials (BCR-2, BIR-1, BHVO-2, RGM-2, GSP-2 and AGV-2) measured by solution ICP-MS from this study and GeoRem preferred values or average published values[59]. The uncertainties (3.8%, 2 RSD) for our measured Nb/Ta are based on six replicates of BHVO-2 (Supplementary Data 1). A 5% uncertainty is assumed for GeoRem data.

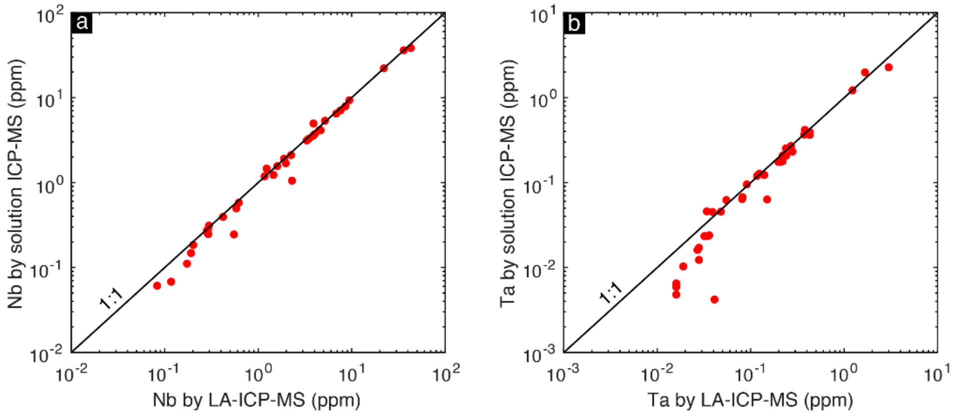

Supplementary Figure 4. Comparison of whole rock Nb and Ta concentrations measured by solution ICP-MS and LA-ICP-MS methods. LA-ICP-MS measurements were done on lithium metaborate-fused glass discs at Rice University. The data were published at http://dx.doi.org/10.1594/IEDA/111138.

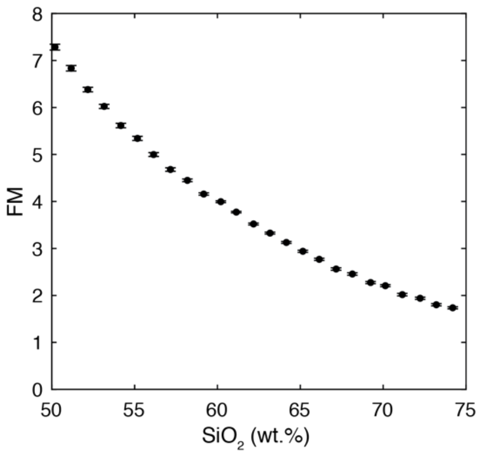

Supplementary Figure 5. Mean magma composition parameter (FM) as a function SiO_2 content in arc magmas. Arc magma composition data are compiled from GeoRoc. The error bars are 2 se and are about the same size as the markers.

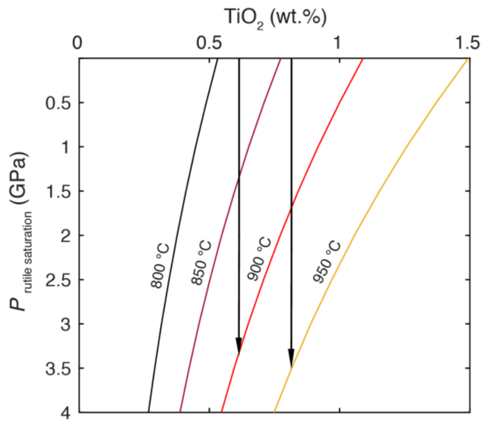

Supplementary Figure 6. Rutile saturation pressure as a function of temperature and TiO_2 content in the melt. We assumed a constant magma composition parameter (FM) of 4, which is the upper bound value for arc magmas with 60−65 wt.% SiO_2 (Supplementary Figure 5).

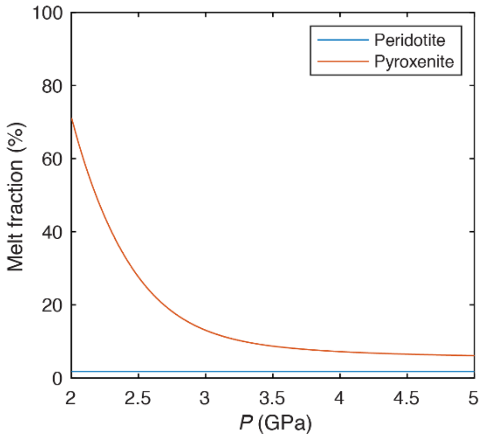

Supplementary Figure 7. pMELTS simulation of garnet pyroxenite and peridotite decompression melting in the mantle.
