# Supplementary material for: Nb/Ta systematics in arc magma differentiation and the role of arclogites in continent formation
Source: Nat Commun. 2019 Jan 16;10:235. doi: 10.1038/s41467-018-08198-3 (PMC6335430; doi:10.1038/s41467-018-08198-3)
Supplement: Supplementary file 3 — Description of Additional Supplementary Files [file 41467_2018_8198_MOESM3_ESM.pdf]

Description of Additional Supplementary Files

File Name: Supplementary Dataset 1

Description: Major elements, Nb and Ta compositions of the reported arclogites; Nb and Ta concentrations in reference materials measured by solution ICP-MS.

File Name: Supplementary Dataset 2

Description: LA-ICP-MS data for rutile, Fe-Ti oxides and sphene measured in this study.

File Name: Supplementary Dataset 3

Description: Compiled dataset for arc, intracontinental and ocean island magmas.
